# Artificial Intelligence in Congenital Heart Disease: Current State and Prospects

**DOI:** 10.1016/j.jacadv.2022.100153

**Published:** 2022-12-14

**Authors:** Pei-Ni Jone, Addison Gearhart, Howard Lei, Fuyong Xing, Jai Nahar, Francisco Lopez-Jimenez, Gerhard-Paul Diller, Ariane Marelli, Laura Wilson, Arwa Saidi, David Cho, Anthony C. Chang

**Affiliations:** a Section of Pediatric Cardiology, Department of Pediatrics, Lurie Children’s Hospital of Chicago, Northwestern University Feinberg School of Medicine, Chicago, Illinois, USA; b Department of Cardiology, Boston Children's Hospital and Harvard Medical School, Boston, Massachusetts, USA; c Division of Pediatric Cardiology, Children’s Hospital of Orange County, Orange, California, USA; d Department of Biostatistics and Informatics, University of Colorado Anschutz Medical Campus, Aurora, Colorado, USA; e Department of Cardiology, Children's National Hospital, Washington, DC, USA; f Department of Cardiovascular Medicine, Mayo Clinic, Rochester, Minnesota, USA; g Department of Cardiology III-Adult Congenital and Valvular Heart Disease, University Hospital Muenster, Muenster, Germany; h Adult Congenital Heart Centre and National Centre for Pulmonary Hypertension, Royal Brompton and Harefield National Health Service Foundation Trust, Imperial College London, London, UK; i National Register for Congenital Heart Defects, Berlin, Germany; j McGill Adult Unit for Congenital Heart Disease Excellence, Department of Medicine, McGill University, Montréal, Québec, Canada; k Department of Pediatrics, University of Florida-Congenital Heart Center, Gainesville, Florida, USA; l Department of Cardiology, University of California at Los Angeles, Los Angeles, California, USA

**Keywords:** adult congenital heart disease, artificial intelligence, cardiac imaging, congenital heart disease, machine learning, outcome prediction

## Abstract

The current era of big data offers a wealth of new opportunities for clinicians to leverage artificial intelligence to optimize care for pediatric and adult patients with a congenital heart disease. At present, there is a significant underutilization of artificial intelligence in the clinical setting for the diagnosis, prognosis, and management of congenital heart disease patients. This document is a call to action and will describe the current state of artificial intelligence in congenital heart disease, review challenges, discuss opportunities, and focus on the top priorities of artificial intelligence–based deployment in congenital heart disease.

## Highlights

• Significant unmet opportunities exist for artificial intelligence to advance congenital heart disease research.
• Leveraging artificial intelligence in congenital heart disease could reduce repetitive tasks and augment clinical decision-making.
• Future research in artificial intelligence should include longitudinal congenital heart disease data to map disease pathophysiology and prognosis across the lifespan.

Artificial intelligence (AI) technologies have made a major impact in imaging in cardiology and have many applications in health care delivery such as computer-assisted diagnostics, risk prediction and stratification, clinical decision support, deep phenotyping, precision medicine, and personalized prescription. Physicians can leverage these to provide optimal care for the patients in the current era of big data.^1^, ^2^, ^3^, ^4^, ^5^, ^6^, ^7^ Congenital heart disease (CHD) is an excellent domain for AI given the robust and diverse data sets extending from complex disease diagnosis and management to multimodality imaging. With evolving therapies and surgeries, CHD patients are surviving longer, creating a growing population of adult CHD patients.^2^ The use of AI could help augment and optimize the management of these patients, improve quality of care, extend life expectancy, save time for the treating physician, and decrease health care costs.

However, there is a significant gap in the application of AI for diagnosis, prognosis, and management of CHD patients across their lifespan. The use of AI in pediatric and adult CHD has been limited by insufficient CHD-specific labeled data sets available for training of models, complex modeling needs in this patient population due to heterogenous clinical phenotypes and age-related pathophysiological changes, and siloed data in center-specific data warehouses.^8^ Additionally, at baseline, data for specific rare forms of CHD are limited, requiring multicentered collaboration to accrue sufficient data sets. Lastly, significant deficits in clinical training, knowledge, experience, and comfort with AI exist.^9^

Despite these challenges, medical intelligence gained from the application of AI technologies and tools to data sets inclusive of the conglomerated CHD population could be instrumental in determining the optimal personalized management strategy for specific lesions. While some AI techniques currently used in adult cardiology may be transferable to adult CHD,^10^ new techniques and collaboration are warranted to address the technical challenges specific to the complexity and rarity of CHD data sets. Therefore, strategic initiatives to promote AI-based research and clinical applications to best serve the unique needs of CHD patients are necessary. This document is a call to action and will describe the current state of AI in CHD, review challenges, discuss opportunities, and focus on the top priorities of AI-based deployment in CHD.

## Basic concepts of AI

**Figure 1:**
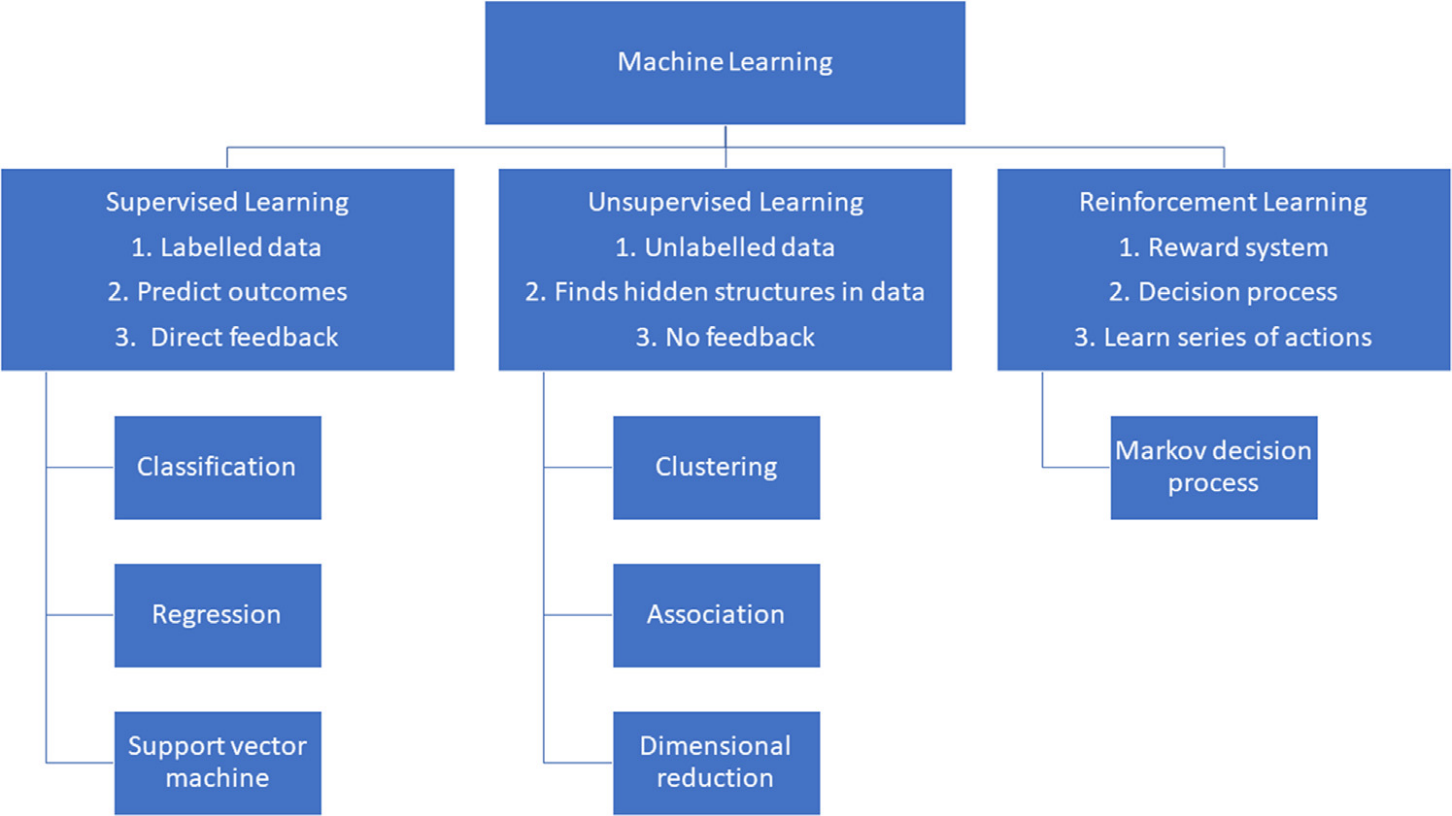
**Different Types of MachineLearning**

AI refers to any technique that enables computers to generate algorithms and find hidden insights to mimic human intelligence. Human intelligence is characterized by the ability to learn, reason, analyze, and make decisions. Machine learning (ML) is a subfield of AI that generates computer algorithms capable of improving task performance by learning or adapting from data.^8^ There are 3 ML strategies (Figure 1): 1) supervised; 2) unsupervised; and 3) reinforcement learning. Supervised learning uses labeled data sets to classify data or perform predictions.^8^ The goal of supervised learning is to learn a function from labelled data sets and produce desired outputs that best describes the relationship between the two.^6^^,^^8^ Unsupervised learning discovers the underlying structure or relationships among variables in an unlabeled data set without dependent variables.^6^ Reinforcement learning is determining the optimal behavior in an environment to earn the maximum reward and is the science behind decision-making.

Deep learning (DL) is a subset of ML that mimics the activity of the layers of neural networks in the neocortex. It has been used extensively in the field of medicine particularly for medical imaging using convolutional neural networks (CNNs), a specific type of deep neural network optimized for image analysis. More recently, CNNs have been applied to cardiovascular data sets for CHD.^1^^,^^11^^,^^12^ CNN models for image analysis are trained using raw imaging data sets and require substantial input data, computational power, and manual labor to label data. Transfer learning is an emerging approach that reduces the computational power needed and allows for faster training of the model. It uses pretrained CNN weights to extract features to apply to the new CNN model to reduce the amount of training data needed to build the model. Another form of DL is recurrent neural networks (RNNs) which use the outputs of some layers of neural network as feedback to use as inputs to the previous layer. This allows for sequential data analysis. A popular framework for learning sequential data is called the long short-term memory network. This is a type of RNN that is capable of learning long-term relationships. Transformers, another type of DL model that learns context and tracks relationships in sequential data, are primarily used in the field of natural language processing and computer vision. RNNs based on long short-term memory or grated recurrent units are capable of learning information dependencies in long input sequences, but they are not parallelizable because the hidden states are computed sequentially. Instead of relying on recurrent structures, transformers process the entire input using a self-attention mechanism, which is easy to be computed in parallel. One potential limitation of transformers is that the computation is very memory-intensive. Transformers in AI are the most recent advancement with implications for CHD clinical work. A generative adversarial network (GAN) is a DL model involving 2 neural networks competing against each other to make more accurate predictions. This type of network is unsupervised and used in image generation.^13^

Recent advances in AI include federated learning and swarm learning frame which can help with data privacy. Federated learning is an ML technique that trains an algorithm across multiple decentralized servers holding local data samples without sharing them.^14^ This type of learning allows the local devices attain the power to learning collaboratively from a shared model. After individual training of models on isolated data sets housed locally, the devices send their specific models to a centralized server where the models are averaged to obtain a single combined model. This process is repeated until a single high-quality model is obtained. Swarm learning is a decentralized, privacy-preserving ML framework that does not rely on a central server.^15^ This type of framework uses the computing power at the distributed data sources to run the ML algorithms that train the model while maintaining data confidentiality.^15^ As such, these systems facilitate data sharing between medical centers. The next section describes the ML and DL models used in CHD.

## Current AI-based pediatric and adult CHD applications and opportunities

Over the last decade, there has been an exponential rise in the number of publications centered on AI in health care, highlighting the potential of this technology. Applications of AI for CHD are robust, ranging from prenatal screening to risk stratification in an aging adult CHD population. In the following section, we review the progress in this field and highlight opportunities to advance unmet needs in areas of prenatal CHD screening, postnatal CHD screening, cardiac imaging processing and interpretation, preprocedural planning, outcome prediction, and precision medicine (Central Illustration).

**Central Illustration:**
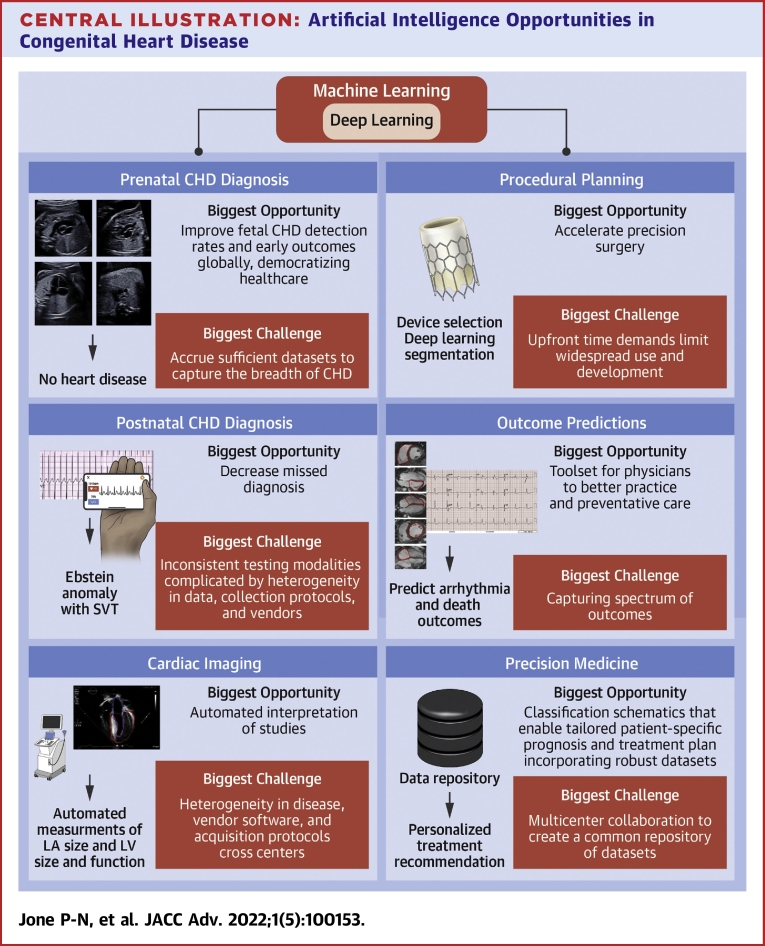
**Artificial Intelligence Opportunities in Congenital Heart Disease** CHD = congenital heart disease; LA = left atrium; LV = left ventricle; SVT = supraventricular tachycardia.

### Prenatal CHD screening

Prenatal screening for CHD can improve neonatal outcomes and offer opportunities for planning in utero therapies, postnatal surgeries, or interventions.^16^, ^17^, ^18^, ^19^, ^20^ Although fetal echocardiography in experienced hands has moderate sensitivity and high specificity reported in the recent meta-analyses, the accuracy of CHD detection is reported as low as 28% in general obstetric practice.^21^^,^^22^ The acquisition of standard cardiac imaging planes is critical in the prenatal diagnosis of CHD, and using AI to automatically retrieve these standard imaging planes from a stream of ultrasound imaging data has the potential to improve CHD detection. The automatically retrieved images may be of higher quality than the manually obtained images. Baumgartner et al^23^ used labeled mid-trimester ultrasound images from 2,694 volunteers and a CNN algorithm to achieve real-time classification of standard-screening fetal cardiac imaging planes (Table 1). Dong et al^25^ used a CNN in 2,032 fetal 4-chamber views and 5,000 views of other fetal structures to evaluate for automatic detection of the 4-chamber view and automatic assessment of image quality. Chen et al^24^ used a composite RNN model in an ultrasound scan of 1,231 ultrasound videos of fetuses to automatically detect standard planes including 4-chamber cardiac views. A landmark article by Arnaout et al^12^ using CNN described the ability to detect complex CHD in utero from normal fetuses. These algorithms can help clinicians and operators who are less experienced in evaluating fetal echocardiograms to detect abnormalities to improve the detection rates of CHD in the community. This could also reduce work time required to obtain normal standard views and allow the sonographer to use the retained time to focus on the evaluation of abnormal cardiac pathology.^47^ Further refinements in AI algorithms or development of fetal CHD-specific learning algorithms could help achieve more granular detections of unique CHD lesions. This has the potential to risk stratify certain fetal populations. Examples of this include improved detection of patients at risk of ductal dependent physiology (ie, coarctation of the aorta) or at risk of intrauterine fetal demise.

**Table 1:** Application of Artificial Intelligence in Congenital Heart Disease

First Author	Year	Patient Population	Category for Analysis	Models	Training/Validation Data Sets	Test Data Set	Results Metrics	Limitations
Prenatal CHD screening								
Chen et al^24^	2017	900 fetuses	Echocardiograms	Composite RNN to define standard fetal cardiac imaging planes	900 videos	331 videos	AUC: 0.95	Limited to healthy patients, not tested on CHD.
Dong et al^25^	2022	3,910 fetuses (14.1% with CHD)	Echocardiograms	Random forest algorithms (ML) to differentiate normal and CHD hearts	25 features	10 features	AUC: 0.94 Sensitivity: 0.85 Specificity: 0.88	Tabular data instead of raw images. No specific subtypes of CHD defined. Single center.
Arnaout et al^12^	2021	1,326 fetuses	Echocardiograms	CNN (classification)	107,823 images from 1,326 echocardiograms	4,108 fetal ultrasounds	AUC: 0.99 Sensitivity: 0.95 Specificity: 0.96	No published algorithms. Not clinically deployed in practice.
Truong et al^26^	2022	3,910 fetuses (14.1% with CHD)	Echocardiograms	Random forest algorithms (ML) to differentiate normal and CHD hearts	25 features	10 features	AUC: 0.94 Sensitivity: 0.85 Specificity: 0.88	Tabular data instead of raw images. No specific subtypes of CHD defined. Single center.
Postnatal CHD screening								
Gharenhbaghi et al^27^	2017	55 healthy children vs 35 BAV	Heart sounds	Support vector machine and Markov model	Unknown	Unknown	Sensitivity: 0.86 Specificity: 0.87	Small study and clinical deployment not widespread.
Gharenhbaghi et al^28^	2020	50 healthy children vs 35 septal defects vs 30 valvular regurgitation	Heart sounds	Time growing neural network (a type of DL)	80 patients for training, 30% random sampling as validation test	Unknown	Sensitivity: 0.92	No test data sets and not used clinically.
Toba et al^29^	2020	1,031 cardiac catheterizations from 657 CHD patients to predict pulmonary-to-systemic flow ratio	Chest x-rays	Transfer learning of CNN	931	100	AUC: 0.88 Sensitivity: 0.47 Specificity: 0.95	Lack of external validation. Bias as all CHD patients who had a cardiac catheterization. Limited number of patients in the training group.
Gomez-Quintana et al^30^	2021	265 term and late-preterm neonates (137 normal vs 89 PDA vs 39 CHD patients)	Heart sounds (healthy vs PDA) (healthy vs CHD)	ML	90% of data	10% of data	AUC (PDA): 0.74 AUC (CHD): 0.78	Not clinically deployed. Limited data sets.
Mori et al^31^	2021	1,192 EKGs from 728 patients (828 normal and 364 ASD)	EKG	CNN and LSTM	Validation was 25% of 1,000 learning data	192 EKG (155 healthy and 37 ASD)	AUC: 0.96 Sensitivity: 0.76 Specificity: 0.96	Volume of data was small for DL. Bias associated with priming effect. Insufficient data to deploy into clinical practice.
Lai et al^32^	2021	236 newborns	Pulse oximetry	ML (random forest, logistic regression, multilayer perception)	158 healthy and 27 CHD patients (0-48 h), 50 healthy and 36 CHD patients (>48 h)	50 healthy and 36 CHD	AUC: 0.91 Sensitivity: 95.8 Specificity: 86.4	Small data sets
Bos et al^33^	2021	2,059 patients; 967 with LQTS and 1,092 evaluated for LQTS but discharged without a diagnosis	EKGs	CNN classification	Trained using 60% and validated in 10% of the patients	Tested on remaining 30% of patients	AUC was 0.900 (95% CI: 0.876-0.925)	Bias as patient cohort sent with suspicion of possible LQTS limiting generalizability. Lacks external validation and calibration from a different center.
Hong et al^34^	2022		Color Doppler echocardiogram images	CNN for classification and segmentation	4,031 cases with 370,057 images	229 cases with 203,619 images of which 105 cases with ASD and 124 with intact atrial septum	Accuracy, recall, precision, specificity, and F1 score of 0.8833, 0.8545, 0.8577, 0.9136, and 0.8546, respectively	Not generalizable to spectrum of CHD; single center.
Cardiac imaging								
Pereira et al^35^	2017	90 patients; 26 coarctation and 64 healthy	2D echocardiograms of the parasternal long axis, apical 4-chamber, and suprasternal notch views	SVM (support vector machine classifiers)	Trained on 80%	Tested on 20%	Total error rate of 12.9% (11.5% false negative error and 13.6% false positive)	Single-center study. Limited to single disease. No external validation.
Diller et al^10^	2019	132 patients with a systemic RV and 67 normal controls (73,425 TGA; 33,394 ccTGA; and 24,354 normal apical 4-chamber frames)	Echocardiograms	CNN—classification and segmentation	159	40	Accuracy: 0.98	Model requires external validation.
Wegner et al^36^	2022	9,793 echocardiogram images from 262 patients with CHD (ToF, Ebstein, TGA) and 62 controls used to build a new model. Prior model was trained on 14,035 echocardiograms from patients without CHD for automated view classification.	Echocardiograms from patients with CHD or structural heart disease used to validate existing CNN trained on structurally normal hearts. Additional model built trained on CHD echocardiograms to compare performance.	CNN view classification model	80% for training and validation	20% for testing	Noncongenital model overall accuracy of 48.3% vs 66.7% in patients without cardiac disease for correct view classification in patients with CHD. New CHD trained model accuracy of 76.1% for view classification.	Single-center study. Not vendor agnostic. Relatively small number of patients with cyanotic forms of CHD (ie, 3 patients with HLHS, 1 with tricuspid atresia).
Karimi-Bidhedi et al^13^	2020	64 patients (20 ToF, 9 DORV, 9 TGA, 8 cardiomyopathy, 9 coronary artery anomaly, 4 pulmonary stenosis, 3 truncus, 2 aortic arch anomaly)	MRI images	Generative Adversarial Network (form of unsupervised learning) to augment data used to augment training set. CNN used to segment MRI images	26 patients randomly assigned to training data set (split 80/20 for training and validation)	38 Patients randomly selected for testing	Dice Similarity Index metrics of 91% and 86.8% for LV at end-diastole and end-systole, respectively, and 87.4% and 80.6% for RV at end-diastole and end-systole, respectively. Externally validated.	Single site. Small patient numbers.
Tandon et al^37^	2021	87 cardiac MRI from repaired ToF patients	MRI images	CNN—transfer learning	57	30	Dice similarity coefficient: 0.90	Small data sets
Wang et al^38^	2021	1,308 children (823 healthy, 209 VSDs, 276 ASDs)	Echocardiograms	CNN view classification for 5 views	90% training	10% testing	Autoencoders trained significantly better on CHD samples than healthy samples; cross-entropy healthy: 0.2649 ± 0.0369 vs 0.2597 ± 0.0327 for CHD, and mean squared difference healthy: 133.89 ± 79.06 vs 118.86 ± 61.52 for CHD. A lower cross-entropy indicates a closer representation of the underlying distribution.	No external validation. Limited diseases.
Procedural planning for catheterization and surgery								
Ruiz-Fernandez et al^39^	2016	2,432 patients	Basic clinical data, healthy history, surgical intervention, and postsurgical intervention	Classification model: 1. Multilayer perceptron 2. Radial basis function 3. Self-organizing map 4. Decision tree	2,432	2,432	Accuracy: 0.99	Not clinically deployed
Lu et al^40^	2020	550 echocardiogram images; 275 before and after atrial septal occlusion surgery	2D echocardiogram images	Variant of the U-Net architecture used to perform atrial segmentation via CNN to determine surgical outcomes of atrial septal defects before and after septal occlude	3:1 Training-to-testing ratio		The U-net mean and SD reported for the Dice Similarity Index, Jaccard Index, and Hausdorff Distance were 0.9488 (±0.0209), 0.9033 (±0.0374), and 7.5625 (±4.4549), respectively.	Single clinical site and scanner used. No external validation.
Outcome prediction and risk stratification								
Diller et al^10^	2019	10,019 adult CHD patients	Clinical data, EKG, cardiopulmonary exercise test, laboratory markers	CNN to categorize diagnostic groups, disease complexity, and New York Heart Association Class	44,000 medical reports	Unclear	Accuracy 91% in diagnosis, 96% in disease complexity, 90% New York Heart Association Class	Retrospective single-center data. Raw echo and MRI data using specifically trained data need validation externally.
Atallah et al^41^	2020	288 patients (72 ToF patients and 216 controls)	Clinical data and noninvasive testing	Random forest Decision tree to risk stratify into low, moderate, high risk for ventricular arrhythmia and life-threatening events	Unknown	Unknown	High-risk group Sensitivity: 0.54 Specificity: 0.86	Small data set and retrospective. Unknown numbers for training and testing data sets.
Jalali et al^42^	2020	549 single-ventricle patients	Clinical data, surgery	Logistic regression Decision tree Random forest Gradient boosting 1. Deep neural network	25 out of 100 variables selected for training	Unknown	AUC (mortality/cardiac transplantation): 0.95 AUC (prolonged length of stay): 0.94	Exclusion of very ill patients from the PHN SVR trial, thus biased toward higher survival rates. Retrospective data set.
Bertsimas et al^43^	2021	235,000 patients with 295,000 operations	Clinical data, general preoperative patient risk factors to predict mortality, postoperative MVST, and length of hospital stay (LOS)	2. Optimal classification trees 3. Random forests 4. Gradient boosting	175,239	46,096	AUC (mortality): 0.86 AUC (prolonged MVST): 0.85 AUC (prolonged LOS): 0.82	Heterogeneous data can lead to bias.
Precision medicine								
Meza et al^44^	2018	651 neonates with critical left heart obstruction	136 echocardiographic measures to group patients into 3 subtypes and identify differentiating characteristics	Unsupervised clustering analysis	Divided into group 1, 215; group 2, 338; and group 3, 98.		Median LV end diastolic area was 1.35, 0.69, 2.47 cm^2^ in groups 1, 2, and 3; *P* < 0.001. Overall mortality was 27%, 41%, and 12%, respectively; *P* < 0.001.	
Bruse et al^45^	2017	60 patients	CMR	Automated segmentation, statistical shape modeling and unsupervised hierarchical clustering to group patients accordingly and identify novel subgroups	Cohort divided into 20 healthy subjects, 20 patients who had undergone surgical aortic arch reconstruction, and 20 patients who had their aorta pushed back posteriorly in the Lecompte maneuver for arterial switch operation		Achieved automatic division of input shape data according to primary clinical diagnosis with an high F-score (0.902 ± 0.042) and Matthews correlation coefficient (0.851 ± 0.064) using the correlation/weighted distance/linkage combination.	Relatively small cohort of patients; not generalizable to other forms CHD
Bahado-Singh et al^46^	2022	24 coarctation patients and 16 controls	Blood spots	Deep learning to perform genome-wide DNA methylation analysis	Unknown	Unknown	AUC: 0.97 Sensitivity: 0.95 Specificity: 0.98	Unknown number of training and testing data sets

ASD = atrial septal defect; AUC = area under the curve; BAV = bicuspid aortic valve; ccTGA = corrected transposition of the great arteries; CHD = congenital heart; CI = confidence interval; CMR = cardiac magnetic resonance imaging; CNN = convolutional neural network; DL = deep learning; DORV = double outlet right ventricle; EKG = electrocardiogram; HLHS = hypoplastic left heart syndrome; LQTS = long QT syndrome; LSTM = long short term memory; LV = left ventricle; ML = machine learning; MRI =magnetic resonance imaging; MVST = mechanical ventilatory support time; PDA = patent ductus arteriosus; PHN = pulmonary hypertension; RNN =recurrent neural network; RV = right ventricle; SD = standard deviation; SVM = support vector machine; TGA = transposition of the great arteries; ToF = tetralogy of Fallot; VSD = ventricular septal defect.

### Postnatal CHD screening

Initial screening in infants for CHD consists of a combination of cardiac auscultation, pulse oximetry, chest radiography, and electrocardiography. A computer-assisted auscultation software program allows the user to store and transmit heart sounds to dedicated platforms for AI-assisted analysis of murmurs.^27^^,^^48^, ^49^, ^50^ Gharehbaghi et al^27^ described using combined support vector machine and hidden Markov models—supervised learning models used for classification—to identify innocent heart murmurs from a bicuspid aortic valve with an accuracy of 86.4%, better than pediatric cardiologists using conventional auscultation (Table 1). This software has not been accepted for clinical use because it was limited by small numbers and lack of widespread clinical deployment.^27^ Furthermore, it was also unclear how the data were split between training and testing data sets.^27^ A subsequent study by Gharehbaghi et al^28^ used a time growing neural network (a type of DL) to differentiate normal heart sounds from systolic murmurs from septal defects or valvular regurgitations. The study population was small, and the test data sets were not used to test the suitability of this methodology as a general clinical tool in the community. Gomez-Quintana et al^30^ used ML on neonatal phonocardiograms to determine the probability of patent ductus arteriosus or CHD in 265 newborns within the first 6 days of life (Table 1). The heart sounds were preprocessed and segmented, then followed by feature extraction. The features were fed into a boosted decision tree classifier to estimate the probability of patent ductus arteriosus or CHD from normal heart sounds.^30^ Finally, the patients were prioritized into the decision of getting echocardiograms to confirm the diagnosis.^30^ This study was the first to identify patent ductus arteriosus using a designed ML-based method and contrast it with an experienced neonatologist's auscultation skills as well as the gold standard of echocardiogram. The model area under the curve was 0.77 for the detection of patent ductus arteriosus.^30^ The authors suggested integrating pulse oximetry to the ML algorithms could improve their framework of a more comprehensive assessment of the performance of AI-augmented decision-making as a clinical decision support tool; however, there has yet to be wide uptake in clinical practice.^30^ Smart stethoscopes have been developed to help with clinical auscultation for detecting CHD heart sounds in remote areas where resources and pediatric cardiology expertise are limited.^51^ Quickly identifying abnormal heart sounds may help triage these patients with appropriate referral for CHD management.^48^ DL has been applied to chest radiographs of CHD patients to aid in the prediction of pulmonary-to-systemic flow ratio (Table 1).^29^ DL has also been applied to electrocardiogram (EKG) readings to detect atrial septal defects.^31^ This DL algorithm that comprised CNN and long short-term memory models may be applicable to other CHD lesions to further aid EKG-based AI diagnostics for the pediatric population.

Wearable technology has gained rapid acceptances into the pediatric community for the analysis of heart rate, blood pressure, oxygen saturation, and heart rhythm. These technologies have the potential of life-saving monitoring in the outpatient setting or remote monitoring for children with CHD and arrhythmias. ML interpretation and prediction using EKG input data are being used for the detection of CHD (Table 1).^31^ The described algorithms for pediatric arrhythmia detection include those for smartwatches^52^ and zio patch^53^^,^^54^ devices. More recently, an ML algorithm built from pulse oximetry features has been created to improve critical CHD detection rates (Table 1).^32^ Future embedding of AI algorithms into the wearable technologies will help to develop connected intelligence, early warning systems, which can be used for prompt risk stratification, targeted early intervention, and personalized prescription. These devices can be used as predictive devices rather than diagnostics.

### Cardiovascular image processing and interpretation

Cardiac imaging such as echocardiography, cardiac magnetic resonance imaging (CMR), and computed tomography (CT) serve as the core of diagnosis and disease surveillance but require significant expertise and time for acquisition and interpretation. DL has been applied to improve each stage of multimodality imaging acquisition and interpretation in adult cardiology (preprocessing, quality optimization, view classification, segmentation, and diagnosis) with less advancement in pediatrics.^55^, ^56^, ^57^, ^58^, ^59^, ^60^, ^61^ This is in large part due to the breadth and subtleties of disease and fewer available training data sets for complex CHD patients, which limits the performance of DL models, increases the chances of overfitting data, and limits the opportunities to externally validate models. Because of this, most published studies focus on specific diseases, making it necessary to implement algorithms not generalizable to the spectrum of CHD into clinical practice. For example, Diller et al^62^ built a CNN algorithm capable of discriminating echocardiograms in adult CHD patients with transposition of the great arteries after the atrial switch, patients with congenital corrected transposition of the great arteries, and healthy controls (Table 1). Although this study had 98% accuracy in identifying CHD, the CNN algorithm has not been externally validated for clinical deployment. Echocardiogram clips have been used to train DL models for the automated diagnosis of atrial septal defects, ventricular septal defects, and coarctation of the aorta.^35^^,^^38^ Furthermore, DL has been used for the segmentation of cardiac structures by ultrasound, CMR, or CT, an essential step to measure anatomic structures and make functional assessments that are integral to disease diagnosis and surveillance. A number of groups have developed strategies to enable DL segmentation algorithms to successfully contour CHD cases using CMR images (Table 1),^13^^,^^37^ echocardiography,^63^ and cardiac CT.^64^ Tandon et al^37^ showed that a CNN algorithm for CMR, developed for structurally normal hearts, was able to be adapted to use in a repaired tetralogy of Fallot (ToF) heart with a relatively small number of training data sets (Table 1). They proposed that similar work can be extended to other forms of CHD.

Automating an otherwise manual step could improve the precision of measurements and efficiency of interpretation. With the implementation of AI, one can reduce the amount of time required in acquiring images, processing images, and reducing variability in interpretation of the images. Computer vision can be leveraged to streamline imaging evaluation and interpretation. By using AI algorithms such as deep neural networks, clinicians can analyze large-volume, nonnumerical data structures such as image processing and apply them to multi-imaging evaluation. This has already been successful in cases of noncongenital cardiac diseases, but there has been limited application in the evaluation of congenital cardiac pathology.^65^ Through the development of CHD-specific learning algorithms, AI could shorten the image-acquisition time, improve image processing, derive interpretation, and facilitate a prompt and precise diagnosis. Future opportunities for AI-enabled echocardiograms from image acquisitions to image interpretation can be developed. In particular, AI algorithms that work with limited labeled data using novel self-supervised and semisupervised approaches will be helpful in CHD.

Implementation of AI in CMR evaluation could significantly benefit the pediatric CHD community. Further development of DL algorithms for CMR reconstruction has the potential to reduce CMR scan time and minimize the effects of motion artifact on imaging quality.^13^^,^^65^, ^66^, ^67^, ^68^ This could have the benefit of minimizing the need of anesthesia for CMR evaluation of young or uncooperative pediatric patients. Furthermore, this could be an opportunity to accelerate fetal CMR research and development. Overall, DL algorithms have the potential to reduce postprocessing time for both the technician obtaining the images and the physician interpreting the study.

### Preprocedural and presurgical planning

Preprocedural planning in catheterization and CHD surgeries requires cardiac imaging integration. GANs (a type of neural network that learns to generate new data from training data sets) have been used successfully to predict the optimal size, shape, and positioning of the transannular patch to optimize outcomes from cardiac CT images of ToF patients.^69^ In a pilot study, cycle adversarial networks were able to align preprocedural CTs with intraprocedural transesophageal echocardiographic images to improve surgical navigation for patients with CHD.^40^ Ruiz-Fernandez et al^39^ optimized AI-based algorithms to improve risk estimation for CHD surgery (Table 1). Deploying AI to automatically segment the pulmonary veins and the left atrium prior to total anomalous pulmonary venous return repair can be crucial for presurgical planning.^64^ Integrating AI into virtual reality in the repair of atrioventricular septal defect using 3D echocardiographic imaging may help with surgical repairs.^70^ These simulated environments for various types of interventions or predictions are potential areas that AI can assist to improve procedural success and outcomes in CHD patients.

### Predictive analytics and risk stratification for outcome predictions

Individualized risk stratification and prognostication in CHD patients often remain challenging and stand to benefit from AI. ML models exist to stratify patients with repaired ToF into low, moderate, or high risk for ventricular arrhythmias using clinical data (Table 1).^41^ Additional models can predict sudden cardiac arrest, ventricular tachycardia, and death using CMR data.^71^ Models for CHD also exist to predict in-hospital mortality,^43^^,^^72^ postoperative complications,^73^ postsurgical bleeding,^74^ prolonged mechanical ventilatory support,^43^ and hospital length of stay.^43^ Raw imaging data sets have been used to predict hemodynamics such as chest film-based estimation of pulmonary-to-systemic flow ratios in CHD (Table 1)^29^ and CT-based prediction of pulmonary pressure after Glenn operations.^75^ ML and DL models have been used to predict and calculate individual patient risk for mortality or cardiac transplantation with high accuracy using the Pediatric Heart Network Single Ventricle Reconstruction trial data set (Table 1),^42^ which might help inform clinical and organizational decision-making.

Risk stratification systems are central to guiding therapy and managing adult CHD patients.^76^ Traditional risk stratification systems in adult CHD have been based on limited data from a single or a limited number of institutions and are usually based on parametric or semiparametric models. The main limitation of these systems is that their models, which have inherent statistical assumptions, have not been validated in real clinical settings. The main promise of AI in the setting of risk stratification in adult CHD is 2-fold. First, due to the more efficient method of data-analysis including raw data directly, AI could facilitate the incorporation of larger or temporal data sets without overburdening available human resources. Second, due to the nonlinearity of AI models, AI algorithms could fit the underlying data more closely, thus improving the predictive ability.^2^^,^^77^ Novel methods for integrating data from remote sites are being developed in AI (ie, federated learning and swarm learning), and this might benefit translational studies in the field.^2^

The feasibility of AI-derived automated risk stratification has been demonstrated. Based on raw medical record data from over 10,000 adult CHD patients, DL algorithms using natural language processing can determine the underlying complexity of disease and predict the need for closer medical attention as well as the risk of mortality in this population.^10^ Furthermore, a study from the German National Register for CHD has shown that AI-based direct risk stratification can be achieved using raw cardiac CMR from patients with ToF. This study is also one of few to assess the external validity of the algorithms by geographically separating training and testing data sets.^71^ Lastly, RNNs represent a promising tool for the analysis of longitudinal medical data in adult CHD patients afflicted by a chronic life-long disease. This innovative method is especially suited to capture long-range nonlinear dependencies such as hospitalizations and future heart failure events. Using the Quebec adult congenital heart disease database, the feasibility of this technique to predict future outcomes and model disease trajectories has been demonstrated.^78^ The aforementioned 3 studies, unlike conventional statistical models, demonstrated that the use of AI algorithms on such large-scale multimodal data sets was critical in risk stratification. While these early data indicate that AI-based models can answer different types of questions compared to conventional statistical models for the development of risk stratification tools,^43^ AI-based models have not been proven to be clearly superior, and conflicting data exist.^79^ With adequate investments and emerging new technologies, novel AI tools are likely to allow for trajectory prediction and inform the optimal timing of interventions.

Advanced analytics leveraging AI show promise to improve patient care. Given the complexity of CHD data, it is difficult to select or design a proper feature representation a priori for specific tasks. By contrast, DL is a representation learning method that directly processes raw input data and automatically learns feature representations in an end-to-end, hierarchical manner. In this way, DL can discover complicated hidden data structures and learn complex transformation functions from CHD data. It also eliminates the need of manual feature engineering that is required in conventional ML techniques. Given the increasing volumes of medical data in the form of text, medical images, and other medical signals, DL models can be developed using the data to provide accurate, clinically relevant predictions in real time. Integrating multiple sources of medical data for a multimodal approach has also enabled prediction of mortality in patients in the intensive care unit.^80^ These multimodal models take advantage of complex inputs such as electronic health record with a wide variety of data such as medical diagnoses, vital signs, prescriptions, and laboratory results to make predictions similar to human clinicians making decisions based on diverse information in clinical practice. Therefore, this multimodal AI approach is a unique opportunity to expand to CHD data sources, which are known to be quite varied.

### Precision medicine

AI in precision medicine involves taking multiple large data points in CHD patients to arrive at a more-accurate diagnosis and specific CHD phenotypes. Big data analytics will help drive the individualized therapy and interventions required for CHD patients. One considerable advantage of AI is its ability to serve as a tool to aggregate and synthesize the many layers of medical data to offer personalized analytics. This includes clinical information, environmental factors, imaging data, and social determinants. Genomic medicine derived from AI will allow for better characterization of the underlying pathophysiology of CHD. Studies have shown the feasibility of CHD screening using serum metabolite panels.^81^ Furthermore, DNA methylation can be used to predict aortic coarctation in neonates (Table 1).^46^ A clustering analysis from unsupervised learning might uncover new subtypes of patients that could benefit from a similar treatment or management. Cainelli et al^82^ applied clustering analysis to children with CHD who had underwent a cardiac surgery to discover 2 distinct profiles: those with a high burden of psychopathology and those with similarities to patients with attention deficit hyperactivity disorders.

Digital twin technology simulates a vision of a comprehensive virtual tool that integrates dynamic clinical data of a patient over time using a mechanistic and statistical model.^83^ The technology serves as a real-time counterpart for a patient and uses mobile health-monitoring data, “omics”, clinical reports, clinical and experimental recordings, and medical images to provide better clinical decision-making and predictions.^84^, ^85^, ^86^ Real-world data can be continuously fed into models to arrive at better prediction outcomes rather than relying on registries or randomized control trials. AI will assist in precision medicine by taking multiple large data points for CHD patients to arrive at more accurate and specific diagnoses of CHD phenotypes. Genomic medicine derived from AI will allow for better characterization of the underlying pathophysiology of CHD. Big data analytics will help drive the individualized therapy and interventions required for CHD patients.

## Challenges in implementing AI in CHD and proposed solutions

Challenges exist in implementing AI in CHD. The first essential step to bringing the potential of AI to reality is to identify significant barriers to integration. Substantial issues related to AI development and integration include the lack of adequate education about AI for clinicians, low volume of data, heterogeneity of data, data imbalance, “explainability” of AI models with interpretability of data, need for collaboration between clinicians and data scientists, and legal barriers. In the following sections, we identify the challenges and propose solutions.

### Lack of AI education for clinicians

The lack of adequate AI education for clinicians poses significant challenges in the clinicians' understanding of AI and subsequent adoption within clinical practice. As with any new modality in medicine, training and understanding of the language used in that modality must be met in order for clinicians to implement this new modality in clinical practice. Without adequate education in AI, it is difficult for clinicians to work with data scientists to create a meaningful clinical project that would be useful for CHD. One proposed solution is introducing AI education in the medical education curriculum and integrating it into the categorical pediatric cardiology fellowship so that the next generation of clinicians will have adequate understanding of AI.

### Low volume of data

Unlike conventional ML models, DL typically requires large data sets for model training. The complexity of CHD and heterogeneity within lesions pose a challenge to collect sufficient data sets that are representative of the breadth of the disease for reliable AI model development. Overfitting algorithms to single-center data sets due to insufficient data and lack of external validation remains a large barrier to gaining physician buy-in, acceleration of research initiatives, and wide implementation of developed algorithms into the clinical environment. Networks for data collection in CHD have been developed, including, but not limited to, ACTION (Advanced Cardiac Therapies Improving Outcomes Network),^87^ FON (Fontan Outcomes Network),^88^ and PROTEA (PartneRships in cOngeniTal hEart disease).^89^ Nonetheless, even these highly curated retrospective data sets are often unable to handle the noisy artifact-laden data generated during patient care. When data are clean and available, such as robust echocardiography data sets, it often requires manual labeling or input for training algorithms, which entails a significant time burden. To overcome a small sample size in CHD, Diller et al^90^ used a strategy by generating 100,000 synthetic images based on CMR data from 303 patients with ToF deemed anatomically plausible by human observers and achieved similar results in comparison to the original patient data. Synthetic data generation, while potentially useful, may lend to modeling bias. Another proposed solution is using GANs or other methods such as sampling for augmenting data from small data sets or some data-insufficient applications. Lastly, some researchers have used transfer learning to overcome data size limits by leveraging model parameters trained on larger data sets. This may be a viable solution in addition to multicenter collaboration.

### Heterogeneity in data and curation

Patients with CHD have a wealth of data sets from wearable devices, intensive care unit stays, and imaging data from multimodality studies. Systems for recording an accurate alignment of events in time might differ by institutional standards. While the abundance of data provides a favorable foundation for algorithmic development, these heterogenous data sets often reside in disparate repositories and formats, creating barriers to access and multicenter collaboration. For example, in CMR, there are different commercialized CMR machine vendors, protocols between hospitals, and varied storage systems. Moreover, a mismatch in data due to a change of environment or disease stage between training and operational data can result in erroneous predictions. This issue has been compounded by the rise of wearable technology with different manufacturers and proprietary data formats. Even in the presence of homogeneity in vendor data sets, hospital infrastructures are poorly equipped for large-scale algorithm development. As a result, multicenter uniform standards and vendor-agnostic model development are necessary to mitigate the heterogeneity of the scanned data.^91^^,^^92^

Creating a CHD consortia, standardizing data with multicenter data collection, and using federated learning are proposed solutions to overcome heterogeneous data sets.^5^^,^^8^ In many circumstances, investigators will have to rely on minimal common data sets, focused on data that are homogeneous across most or all centers, to base the core of the analysis on these data, while using data that are more subject to variability only for secondary analyses. Moving forward, it will be important to increase the standardization of study protocols, data collection, and the homogenization of the definition of clinical findings and symptoms, ideally collected with standardized forms. For example, the use of digital imaging and communications in medicine has facilitated the standardization of imaging in medicine, as standard formats for EKG raw signals have facilitated multicenter AI research using EKG.^93^^,^^94^ Similarly, the investigators will need to identify ways to make electronic health record interoperable, something that will facilitate the collection of clinical data from multiple centers.

### Data imbalances and bias

Data sets in CHD, particularly medical imaging information, are subject to imbalances in representation that can be perpetuated by AI development. For example, CMR data sets contain primarily abnormal subjects with very few normal cases, and the opposite is true for echocardiography. Small and single-center data sets can lead to skewed and overfitted training data sets to specific populations, limiting applicability to real-life scenarios such as patients of different races or socioeconomic classes.^95^^,^^96^ Underrepresentation in available data sets may limit access to AI-driven solutions, compounding health care inequity in the CHD population.^97^^,^^98^

To address the issue of data imbalance, the CHD community will need to create prospective data sets of normal children in research setting to have a point of comparison for when the algorithm requires normal controls for echocardiograms, EKGs, CTs, or other cardiac testing. ML solutions will also need to be tested rigorously in different settings and populations so equity and the potential for bias can be continuously monitored after implementation.

### Evidence and “explainability”—trust

The high stakes of managing CHD warrant evidence and “explainability” of research, particularly to overcome the reluctance among clinicians unfamiliar with AI to adopt the technology in clinical practice. The majority of clinicians are experienced with traditional and transparent health care research models. In contrast, in AI model development, algorithms may be programmed to arrive at the output without clear instructions; the infamous “black box” problem. The inability of the AI system to explain how it arrives at the prediction is a serious technical challenge that prevents trust from clinician users. Furthermore, reports of publicly available AI tools potentially causing harm to patients display the potential downfalls of AI-based solutions used without appropriate validation. As such, clinicians and the public will likely mandate a degree of “explainability” before AI integration. It is also unlikely that clinicians will trust the AI model if it does not give correct predictions.

To address the “explainability” issue, computer scientists are currently working on methods to identify the key explanatory variables, in order to decode the “black box.” Those efforts will help to ease the concerns of knowing exactly how the computer identifies specific conditions or predicts outcomes. However, some degree of uncertainty in how the AI-based solution works will have to be accepted. This is akin to the number of medications used in clinical practice for decades which have no clear or known mechanism of action but are used routinely. To this end, as long as the AI-based solutions prove to be safe and effective to detect conditions or predict outcomes, clinicians, patients, and the public in general will become more tolerant to the relative uncertainty to understand how the machine works.

### Need for collaboration between clinicians and data scientists

A major challenge in integrating AI with medicine has been the disconnect between clinical investigators and computer scientists in terms of what is important for patients, the definition of problems, and ways to solve them using AI. To address this, engineers and computer scientists will need to become more familiar with clinical practice and to see firsthand the potential AI-based solutions that would benefit clinicians and patients. At the same time, clinicians and scientists without prior experience in computer sciences will need to embrace new information and skills to better understand the way ML is developed. Over time, people from both sides will also need to appreciate that the terminologies that are often named differently actually refer to the same concept. For example, what clinical investigators call variables, computer scientists call features, and what clinical investigators call outcomes, engineers call labels. Building bridges in education between both fields is imperative to accelerate the development of clinically useful tools.

### Liability and legal concerns

Members of the hospital legal system are often unfamiliar with how to address accountability and liability should health care professionals utilize AI in practice. Incorrect predictions made by AI algorithms can result in severe, lifelong consequences for patients, requiring a high degree of caution, oversight, and quality control. Ambiguity in terms of intellectual property and who ultimately owns the data (ie, patients, the hospital, or developers) may also complicate the milieu. As with any innovation, legal corollaries regarding the use of patient-generated data for partnerships with industry to develop AI-based systems must be determined. Currently the American Medical Association and the Food and Drug Administration are independently working on defining major ethical and legal dilemmas brought by AI in medicine. It is expected that national scientific societies, federal agencies, and other organizations will come up with clearer guidelines addressing those potential legal and ethical dilemmas in the near future.

## Call to action in CHD

A call to action to broaden the expansion of AI in CHD requires multicenter collaboration, curation and creation of data sets, building institutional AI infrastructure, and implementing AI best practice and AI education and training.

### Multicenter collaboration

Multicenter collaboration is necessary to accrue large data sets to train AI algorithms. Multicenter registries have been started. These deidentified data sets can be housed in the cloud so that there is a common repository of these CHD lesions for multiple centers to access to produce meaningful AI solutions. As such, expansion of training data sets with rare CHD cases can be achieved. This would allow for dissemination of expertise in diagnosis and management of CHD lesions.^99^ The feasibility of progressive GANs has been demonstrated in CMR.^90^ Collaboration between AI clinicians, computer scientists, and administrations are important for each center to participate in multicenter collaborations.

### Creation and curation of data sets

Creating and curating data sets to provide high-quality data to train ML algorithms will result in acceptable generalizability when external validations are performed. Data sets will need to be labeled with specific CHD lesions for supervised learning. Unsupervised learning may result in clusters of information that we have not seen in the data sets although the noise ratio maybe quite high. Data sets that are from real world and multimodal will create opportunities for AI in CHD. The advantage of ML in terms of data integration is that it solves problems when there are large amounts of features that are available, and it allows for integration of diverse types of data. Future states of unsupervised learning, semisupervised learning, and transfer learning may help with reducing the need for curation of data.^100^ Data sharing will be less of an issue with federated and swarm learning in the future.

### Building institutional AI infrastructure

Building institutional AI infrastructure requires stakeholders who are interested in AI implementation in pediatric and adult cardiology, hospital IT systems to support data and AI algorithms, and integration of AI process into the clinical workflow (Table 2). AI champions must work with data scientists and hospital leadership with vested interest in AI projects designed for CHD. A regulatory process must be established to allow for AI projects with the hospital IT support to test and retest AI algorithms so that it is clinically valid. Once the validity of the AI algorithm has been achieved, these AI algorithms may be embedded into clinical workflow to enhance clinician's efficiency and workflow.

**Table 2:** Barriers to Artificial Intelligence Implementation in Congenital Heart Disease and Proposed Strategies to Overcome Them

1. Insufficient data access, storage, and sharing strategies for CHD patient data. Data limitations are due to lack of accurately labelled data. Methods such as transfer learning, self-supervised learning, and predictive learning to increase these data may help overcome these barriers to increase opportunities for external validation
2. Lack of AI in medicine awareness from stake holders in health care (ie, clinicians, patients, and hospital administrators). Clinicians need more education about data and AI, and patients need more education to understand the need for and benefits of collaboration on real-world data and not just registries and randomized control trials. Developing institutional educational series and profession society webinars (American College of Cardiology Innovation and Adult Congenital and Pediatric Cardiology sections) may help address these challenges.
3. Absence of forums to facilitate communication between clinicians and data scientists. Providing computer and data scientists with more knowledge regarding the proposed deficits in health care to target the development of meaningful AI solutions. Increasing clinician-to-data scientist synergy for mutual understanding of the dual perspectives of both domains.
4. Difficulty harnessing collaboration. Recruitment of multidisciplinary team members, particularly AI champions, to drive AI implementation.
5. Current CHD research is unidimensional. Leveraging multimodal AI for cardiology to incorporate the full spectrum of data: genomics, imaging, demographic, ICU, wearable, and so on to accelerate precision medicine.
6. Concern that AI methods are not transparent enough for the medical community. Utilizing explainable AI to minimize the “black box” perception of AI and requiring studies provide documentation that they completed the recommended Minimum Information About Clinical Artificial Intelligence Modeling Checklist.
7. Critique that AI projects are not created in the context of clinical applicability. Utilizing design thinking to select proper AI methodology relevant to the clinical context.
8. Poor acceptance of AI in the research community and concern that AI requires too much time to establish sufficiently large data sets. Using innovative AI methods to leverage the power of small data sets. Executing more realistic projects that are easier to accomplish, with demonstrable value and return on investment (ROI) may help get “buy-in” from the administrative and clinical leadership.

AI = artificial intelligence; CHD = congenital heart disease; ICU = intensive care unit.

### Implementing AI best practices

AI best practices starts first with high-quality data to ensure that the model derived from these data sets can be applied to test data sets with good performance. Second, the model's performance should be reproducible so that it can be easily replicated and applied to the subgroup analysis and external validation data sets. Third, the model must have good generalizability and oversight from clinicians so that there are no unintended consequences to the patients or the health care system. Lastly, the model when applied to patients or a clinical problem must be clinically relevant and consistent with clinical judgement or guidelines.

### Education and training

Clinician education and training in different modalities of AI is important to facilitate communication with data scientists. Clinicians and data scientists must work together on AI projects to answer the clinical questions that will impact patient care. Without the clinicians' input in what is clinically meaningful, the models generated may not be helpful. Clinicians will also need to ensure the accuracy and functionality of AI-powered results through testing within the clinic. Data scientists will need to learn to develop AI models relevant to addressing clinical questions; in this regard, it would benefit data scientists to collaborate with clinicians so that the former can better understand the workflow of clinical practice and how AI-based solutions can be integrated into the workflow.

### CHD areas for implementing AI: top priorities

Key areas to focus in future AI research and deployment in CHD include: 1) targeting rare diseases (coronary artery anomalies); 2) acquired diseases that disproportionately affect patients with limited access to pediatric cardiologists for disease diagnosis (rheumatic heart disease); 3) congenital heart anomalies with high morbidity and mortality (failing Fontan or ToF patients at risk of sudden cardiac death); and 4) precision medicine for decision-making in difficult diseases (borderline left heart). Randomized control trials are difficult to perform in CHD because of the disease complexity, disease rarity, and clinical heterogeneity of different lesions. Together, these decrease the precision of the treatment options for patients as most CHD treatment recommendations are based on expert consensus. Collaborative AI research focused on aggregating data and sharing insights into rare diseases that could help clinicians with better decision-making.

Some areas of urgent need for AI research have been explored and contain ample opportunities for future collaboration and extension. For example, Meza et al^44^ used unsupervised ML to identify patterns in echocardiographic data that could be clinically relevant to diagnosis and prognosis of patients with borderline left ventricle. In this study, parameters like mitral valve characteristics and pulmonary vein anomalies that are often used in clinical practice to help guide management in the borderline left ventricular condition were not found to be significant in distinguishing patients from 3 different groups (multilevel left ventricular hypoplasia, hypoplastic left heart syndrome, and critical aortic stenosis). Diller et al^71^ developed an automatic DL imaging algorithm that predicted death/aborted cardiac arrest and documented ventricular tachycardia in ToF patients. Other applications such as the prediction of the feasibility of and risk associated with surgical or catheter-mediated interventions^42^^,^^43^^,^^69^^,^^72^^,^^101^ and individualized prediction of drug effects or interventions in complex hemodynamic settings show promise for future CHD extension.^86^

With the advent of increased computing power, clinicians can leverage AI for precision medicine with better clinical decision-making, and patients can receive real-time information about their personal health metrics. With increased cognitive computing with natural language processing, reinforcement learning, and DL, AI will have better future prediction models and drug therapeutics in patients with CHD. Digital twin will provide cardiologists the best therapy without relying on published reports or registries in the future.

## Conclusions

The unique strength of AI models is the uncanny ability to learn from data with increased exposure. Leveraging AI to accelerate and strengthen CHD research and clinical applications is now possible largely due to the escalating volume and complexity of data available and advent of increased computing power. Clinicians and patients could soon benefit from clinical decision-support tools that assist with personalizing patients' diagnosis, prognosis, and treatments and provide real-time information on personal health metrics. Although there are challenges in the implementation of AI in CHD, opportunities exist in many areas of CHD for clinicians to explore. With the arrival of newer AI-powered algorithms capable of handling big data, such as DL using CNN and RNN, federated learning, and digital twin, a vast amount of research opportunities exist to collaborate and study CHD across a lifespan to build future prediction models and develop drug therapeutics in patients with CHD.

## Funding support and author disclosures

The authors have reported that they have no relationships relevant to the contents of this paper to disclose.
